# Drivers of body condition in South American sea lion pups along a latitudinal gradient

**DOI:** 10.1093/conphys/coag018

**Published:** 2026-03-30

**Authors:** Alicia I Guerrero, Carola Vivanco, Guido Pavez, Pablo Sabat, Karin Maldonado, M Fernanda Barilari, Renato A Quiñones, Pablo Carrasco, Frederick Toro, Josefina Gutiérrez, Maritza Sepúlveda

**Affiliations:** Centro de Investigación y Gestión de Recursos Naturales (CIGREN), Facultad de Ciencias, Universidad de Valparaíso, Gran Bretaña 1111, Playa Ancha, Valparaíso, 2360102, Chile; Centro de Investigación y Gestión de Recursos Naturales (CIGREN), Facultad de Ciencias, Universidad de Valparaíso, Gran Bretaña 1111, Playa Ancha, Valparaíso, 2360102, Chile; Centro de Investigación y Gestión de Recursos Naturales (CIGREN), Facultad de Ciencias, Universidad de Valparaíso, Gran Bretaña 1111, Playa Ancha, Valparaíso, 2360102, Chile; Gestiona Consultores, Francisco Noguera 200, Of. 1201, Providencia, Santiago, 7500006, Chile; Departamento de Ciencias Ecológicas, Facultad de Ciencias, Universidad de Chile, Las Palmeras 3425, Ñuñoa, Santiago, 7800003, Chile; Center of Applied Ecology and Sustainability (CAPES), Av. Libertador Bernardo O'Higgins 340, Santiago, 8331150, Chile; Millenium Nucleus of Patagonian Limit of Life (LiLi), Universidad Austral de Chile, Independencia 631, Valdivia, 5110566, Chile; Departamento de Ciencias, Facultad de Artes Liberales, Universidad Adolfo Ibáñez, Diagonal las Torres 2640, Peñalolén, Santiago, 7941169, Chile; Centro de Investigación y Gestión de Recursos Naturales (CIGREN), Facultad de Ciencias, Universidad de Valparaíso, Gran Bretaña 1111, Playa Ancha, Valparaíso, 2360102, Chile; Departamento de Oceanografía, Interdisciplinary Center for Aquaculture Research (INCAR), Universidad de Concepción, Barrio Universitario s/n, Concepción, 4030000, Chile; Departamento de Oceanografía, Interdisciplinary Center for Aquaculture Research (INCAR), Universidad de Concepción, Barrio Universitario s/n, Concepción, 4030000, Chile; Escuela de Medicina Veterinaria, Facultad de Recursos Naturales y Medicina Veterinaria, Universidad Santo Tomás, Av. 1 Norte 3041, Viña del Mar, 2561694, Chile; Programa de Doctorado en Ciencias mención Ecología y Evolución, Facultad de Ciencias, Universidad Austral de Chile, Independencia 631, Valdivia, 5110566, Chile; Centro de Investigación y Gestión de Recursos Naturales (CIGREN), Facultad de Ciencias, Universidad de Valparaíso, Gran Bretaña 1111, Playa Ancha, Valparaíso, 2360102, Chile

**Keywords:** Bergmann’s rule, body condition, Chile, maternal effect, net primary productivity, pinnipeds

## Abstract

Body condition is a key proxy of fitness in pinnipeds, reflecting nutritional status and maternal investment. In the South American sea lion (*Otaria flavescens*), pup growth and survival depend on maternal foraging success, making pup condition a sensitive indicator of local environments. We quantified spatial and interannual variation in pup body condition across five Chilean breeding colonies spanning 21–53°S during the austral summers of 2024 and 2025. We captured 157 live pups (95 males, 62 females), measured morphometrics and calculated a body condition index (BCI = mass/length). To account for seasonal effects, BCI values were standardized to allow comparisons across sites and years. We tested the effects of sex, year, locality and satellite-derived net primary productivity (NPP). Male pups consistently showed higher standardized BCI than females. Locality was the strongest predictor: Isla Marta (southern limit) exhibited significantly higher values than all other sites, followed by Isla Metalqui. Cobquecura, Isla Choros and Punta Lobos showed lower or intermediate values. Year alone had no effect, but a significant locality × year interaction indicated interannual variability in northern colonies, particularly Punta Lobos. NPP was not retained in top-ranked models, suggesting broad-scale productivity does not directly predict pup condition at this resolution. The pronounced latitudinal gradient, with larger, better-conditioned pups at higher latitudes, is consistent with expectations under Bergmann’s rule, which refers to the tendency of animals to be larger in colder climates and smaller in warmer ones. These results underscore the combined influence of local ecological conditions, maternal effects and intrinsic sex differences on pup condition and reinforce the value of South American sea lion pups as sentinels of ecosystem variability along the Chilean coast.

## Introduction

Body condition is an indicator of an animal’s nutritional status and energy reserves, with individuals in better condition generally having greater energy stores (typically fat) than those in poorer condition (Schulte-Hostedde *et al.*, 2005; Labocha *et al.*, 2014). This parameter is a key variable in many ecological studies, as a positive correlation has been found between body condition and both survival and reproduction across various taxa (e.g. Guinet *et al.*, 1998; Blums *et al.*, 2005; Devries *et al.*, 2008; Morgan-Davies *et al.*, 2008; Stewart *et al.*, 2021; Wachtendonk *et al.*, 2022). Body condition can exhibit spatial and temporal variability driven by environmental and ecological factors, with local productivity being a major determinant through its influence on resource availability. In marine environments, where productivity varies across both spatial and temporal scales, it strongly influences the distribution, diet, foraging behaviour, diving patterns and body condition of marine predators (Sepúlveda *et al.*, 2014).

Among marine predators, marine mammals are widely recognized as sentinel species, i.e. organisms whose health, condition or behaviour can provide early warnings of environmental change (Fossi *et al.*, 2017). This is largely due to their long life spans, long-term residency and position as top predators, which make them sensitive to shifts in food web dynamics (Bossart, 2011). In addition, their large lipid reserves can accumulate persistent pollutants, providing long-term information on ecosystem health (Jepson and Law, 2016). Otariids (fur seals and sea lions) are particularly suitable indicators of changes in marine productivity, since most species are central place foragers and income breeders that exhibit strong coastal residency and philopatric behaviour, with females typically foraging near their reproductive colonies (Costa and Valenzuela-Toro, 2021). Such foraging strategies make them especially responsive to local prey fluctuation, directly linking prey availability and fitness (Womble *et al.*, 2009; Sepúlveda *et al.*, 2014).

Marine productivity along the Chilean coast (18° to 56°S) exhibits strong latitudinal variation (Daneri *et al.*, 2000), shaped by upwelling dynamics, coastline features and freshwater inputs (Montecino and Lange, 2009). The northern and central regions (18–37°S) represent one of the most productive sectors of the Humboldt Current System, with persistent wind-driven upwelling sustaining extremely high primary production and supporting major small pelagic fisheries (Thiel *et al.*, 2007; Montecino and Lange, 2009). In contrast, productivity in south-central Chile (37–40°S) is more seasonal (Aguirre *et al.*, 2021), while in the Patagonian fjords (41–56°S), it is generally lower and more heterogeneous, with localized spring–summer blooms influenced by river discharge and estuarine circulation (Iriarte *et al.*, 2007). This latitudinal variation in marine productivity is likely to impact marine predators with strong dependence on local prey availability, such as the South American sea lion (*Otaria flavescens*).

Like other otariids, South American sea lions rely heavily on local prey availability, showing pronounced latitudinal shifts in diet composition in response to prey abundance variability (Muñoz *et al.*, 2013; Guerrero *et al.*, 2020). Throughout the austral summer breeding season (January–February) and the subsequent non-breeding period (March–December), adult females act as central-place foragers during the entire lactation period (7–12 months; Crespo *et al.*, 2021). They alternate short foraging trips at sea with periods on land to nurse their pups (Muñoz *et al.*, 2013). These trips typically last 1–2 days, with females usually remaining within 50–100 km of the rookery (Sepúlveda *et al.*, 2015; Frantz *et al.*, 2024). Because pup growth and survival depend almost entirely on the mother’s ability to secure sufficient food resources during both gestation and lactation (Lea *et al.*, 2006; Costa and Valenzuela-Toro, 2021), and given the pronounced latitudinal variability in marine productivity along the Chilean coast, mean pup body condition at the colony level is expected to vary spatially in response to fluctuations in the distribution and abundance of prey during the nursing period. Consequently, spatial differences in average pup body condition among colonies may serve as a sensitive indicator of how changes in prey quantity and quality affect different South American sea lion populations. This in turn has important demographic implications, since in otariids, pup body mass and body condition have been shown to be positively associated with early survival. For instance, in Steller sea lions, pups with higher body mass and better body condition show higher survival probabilities during the first months of life (Pendleton *et al.*, 2016). More generally, variation in maternal foraging success and resource allocation can translate into differences in pup growth, physiological condition and immune competence, ultimately affecting offspring survival, as shown for South American fur seals (Perez-Venegas *et al.*, 2024). Conversely, poor pup condition may reflect nutritional or environmental stress (Oosthuizen *et al.*, 2019) with potential negative consequences for early-life performance and survival.

Body condition may vary due to spatial differences in marine productivity, as well as sex-related differences in milk intake, growth rate and energy allocation during lactation. Several studies have reported that male pups are generally larger than female pups, which has been attributed to higher milk intake and faster growth rates in males (Keogh *et al.*, 2013). According to Kraus *et al.* (2013), the higher sensitivity of male pups to harsh environmental conditions may lead mothers to favour male offspring to enhance their survival prospects. However, other studies have found no significant sex differences in either milk intake or growth rates (Lunn and Arnould, 1997), suggesting that sex-specific patterns may vary across populations or environmental contexts. These discrepancies highlight the importance of considering both intrinsic (e.g. sex, metabolic demands) and extrinsic (e.g. food availability, maternal foraging success) factors when evaluating pup body condition. This is particularly relevant in species with prolonged lactation periods (i.e. >7 months; Crespo *et al.*, 2021), such as the South American sea lion, where maternal investment and environmental variability interact to shape pup survival and fitness. Thus, pup body condition is not only an individual trait but also a proxy of maternal foraging success and local ecosystem variability.

Considering the pronounced latitudinal gradient in marine productivity along the Chilean coast, we hypothesize that spatial variation in South American sea lion pup body condition is primarily driven by regional differences in productivity. Based on this hypothesis, we predict that pups from highly productive regions will exhibit better condition than those from less productive areas. Accordingly, the aims of this study were to: (i) evaluate whether the body condition of South American sea lion pups differs among colonies distributed along a latitudinal gradient of the Chilean coast, (ii) assess whether body condition varies between male and female pups within these colonies and (iii) examine the extent to which spatial and temporal differences in prey availability influence pup body condition across years and localities.

## Materials and Methods

### Study area

**Figure 1:**
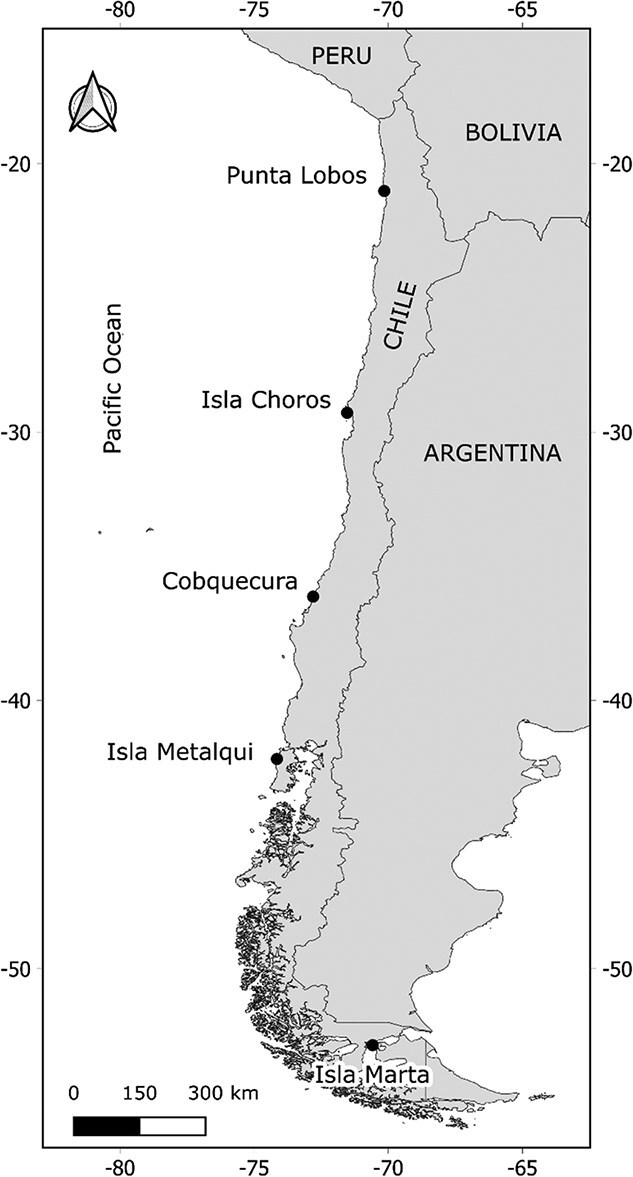
Map of the study area showing the five South American sea lion (*O. flavescens*) breeding colonies sampled along the Chilean coast.

Data were collected during the austral summers of 2024 and 2025 in five South American sea lion breeding colonies along the Chilean coast: Punta Lobos (21°01′S; 70°09′W), Isla Choros (29°17′S; 71°32′W), Cobquecura (36°08′S; 72°48′W), Isla Metalqui (42°11′S; 74°09′W) and Isla Marta (52°51′S; 70°35′W) (Fig. 1). Punta Lobos is a breeding colony located in northern Chile, with an estimated population of 560 individuals (Oliva *et al.*, 2020). Isla Choros, part of the Choros-Damas Marine Reserve, hosts ~350 individuals (Oliva *et al.*, 2020). Cobquecura is a rocky island situated ~80 m off the central-southern Chilean coast and represents the most important breeding site for South American sea lions in central Chile, with an estimated population of 3200 individuals (Sepúlveda *et al.*, 2014; Oliva *et al.*, 2020). Isla Metalqui, a large island on the exposed coast of Chiloé, comprises four distinct beaches and is the most important South American sea lion breeding colony in the country, supporting an estimated 19 600 individuals (Oliva *et al.*, 2020). Finally, Isla Marta, located at the southernmost limit of the species’ distribution in Chile, harbours ~510 individuals (Venegas *et al.*, 2002). This colony lies within the Strait of Magellan, a transitional environment influenced by the mixing of Pacific and Atlantic waters, fjord systems and strong tidal currents. These unique oceanographic conditions sustain high local productivity and may provide distinctive foraging opportunities for lactating females.

The pupping season of the South American sea lion along the Chilean coast occurs during the austral summer months of January and February, with a peak in births during the second half of January and the first half of February (Acevedo *et al.*, 2003). By the end of the breeding season (second half of February), reproductive activity ceases and the number of adult males in the colonies gradually declines (Sepúlveda *et al.*, 2001). As pups are not yet capable of swimming long distances, females remain with their offspring within the colony until approximately May, when pups become more proficient swimmers and females begin to leave the breeding sites (Sepúlveda *et al.*, 2009).

### Capture and measurements of South American sea lion pups

A total of 157 live pups (95 males and 62 females) were randomly captured in 2024 (*N* = 89) and 2025 (*N* = 68) across the five colonies (Table 1). In all colonies—except Cobquecura—three to four observers captured individuals directly within the colony. Each pup was captured using a hoop net made of fine-mesh nylon, with a 1-m-deep and 80-cm diameter bag. To minimize disturbance, most fieldwork was conducted after February 25, once the breeding season had ended and most adult males had left the colonies (Sepúlveda *et al.*, 2001), reducing the likelihood of stampedes, which could endanger pups. Accordingly, individuals were captured between late February and March at all sites, except for Isla Marta in 2024, where, due to unfavourable environmental conditions, pups were sampled in mid-April.

**Table 1:** Number of South American sea lions (*O. flavescens*) pups captured in five colonies along the Chilean coast during the summer of 2024 and 2025

**Colony**	**2024**		**2025**	
	**Males**	**Females**	**Males**	**Females**
Punta Lobos	5	11	7	0
Isla Choros	9	6	11	4
Cobquecura	16	10	7	9
Isla Metalqui	10	6	8	7
Isla Marta	11	5	11	4
Total	51	38	44	24

All procedures involving animals were reviewed and approved by the Institutional Committee for the Care and Use of Laboratory Animals of the Universidad de Valparaíso (CICUAL-UV; protocol BEA196-23).

In Cobquecura, high waves frequently wash healthy pups out of the colony during the breeding season (Sepúlveda *et al.*, 2014, 2020). Therefore, during the second half of January each year, two observers were positioned in front of the colony to capture live pups immediately upon their stranding on the beach. As described by Sepúlveda *et al.* (2014), we assumed that all pups had a similar probability of stranding, given that high waves regularly impact different areas of the colony (Sepúlveda *et al.*, 2020).

Each pup was sexed, measured [including standard length (L; straight-line distance from nose to tail in dorsal view) and axillary girth (in dorsal view)] and weighed to the nearest 0.1 kg using a spring scale. After all procedures were completed, each pup was marked with water-resistant paint on one fore flipper to prevent repeated measurements of the same individual and was subsequently released.

### Marine productivity

Net primary productivity (NPP) data were obtained from monthly satellite images derived from the Eppley Vertically Generalized Production Model based on VIIRS sensors (NASA Earth Sciences, 2020). NPP was used as a broad-scale, bottom-up proxy of primary productivity and potential energy input to higher trophic levels (Ware and Thomson, 2005). Because there can be a time lag between peaks in phytoplankton production and peaks in prey availability for lactating otariids (Suchy *et al.*, 2022), satellite-derived NPP should be interpreted as an indirect indicator rather than a direct measure of prey availability at the colony scale.

Data were downloaded in HDF format and subsequently converted to GeoTIFF in the WGS84 projection. Each file was reprocessed in R using the *terra* package (Hijmans *et al.*, 2022), defining the global extent and correcting spatial orientation. Monthly layers spanning 2014–2023 were then stacked and averaged to generate a raster representative of mean decadal NPP. Data availability extended only through December 2023.

To avoid bias in the calculations, negative NPP values were set to zero, assuming that they represented either undetectable levels of production by the sensor or numerical artefacts from satellite processing. For each site of interest, NPP values were calculated by averaging raster cells within a 40-km buffer radius around each South American sea lion colony. This radius was chosen to approximate the lower bound of the spatial scale of maternal foraging trips during early lactation, as females generally forage within a few tens of kilometres from their breeding colonies (Riet-Sapriza *et al.*, 2013; Sepúlveda *et al.*, 2015). For the rookery at Isla Marta, precise NPP data were not available because the colony is located within the fjord system of the Strait of Magellan, where the satellite product lacks sufficient spatial resolution. Therefore, NPP values were extracted from the nearest offshore area with reliable data coverage, located at the eastern entrance of the Strait of Magellan (Bahía Posesión, Atlantic side), ~80 km from the colony.

### Data analyses

To account for seasonal variation in pup body condition throughout the breeding period, we applied a correction based on historical data from the breeding colony of Cobquecura, central Chile (here on, the reference colony). The dataset included body mass and standard length measurements from 834 South American sea lion pups sampled between 2009 and 2020, and spanning over 80 days from the start of the breeding season. Although the reference colony of Cobquecura was also included among the localities surveyed in the present study, the historical dataset was used exclusively for model calibration. For each pup, we calculated a body condition index (BCI) as the ratio of body mass (kg) to standard length (cm), a widely used proxy for relative condition in pinnipeds (Bradshaw *et al.*, 2000). Although alternative approaches, such as regression residuals or the scaled mass index (Peig and Green, 2009), have been proposed, the mass/length ratio remains a robust and practical metric for field studies in pinnipeds.

To verify that seasonal changes in BCI were not a geometric artefact of mass–length scaling, we examined the allometric relationship between body mass and length using the >10-year historical dataset. The log–log regression revealed a strongly sub-linear scaling (exponent = 0.84), clearly ruling out volumetric growth over this size range. This confirms that the observed seasonal increase in BCI reflects a real biological change in body condition rather than a mathematical consequence of size scaling.

To describe the seasonal trend in BCI, we fitted a linear mixed-effects model with BCI as the response variable, day of the breeding season (“Progression”) as a fixed effect and sex as a random intercept to account for baseline differences between males and females. In this analysis, January 1 was defined as day 1 of the breeding season.

The model provided an estimate of the expected daily change in BCI (*β₁*) over the course of the season, controlling for sex. Using this estimate, all BCI values in the current 2024–2025 dataset were standardized to a common reference date corresponding to the latest sampling day in the season. The adjustment followed the equation:


$$ {\mathrm{BCI}}_{\mathrm{standardized}}={\mathrm{BCI}}_{\mathrm{obs}\mathrm{erved}}+{\beta}_1\ast \left({\mathrm{Progression}}_{\mathrm{ref}}-{\mathrm{Progression}}_{\mathrm{obs}}\right) $$

where Progression_obs_ and Progression_ref_ represent the number of days elapsed since January 1 for the observed and reference sampling dates, respectively. This procedure increased BCI values for pups measured earlier in the season, while those measured on the reference date retained their original values, ensuring comparability across sites regardless of sampling timing.

After adjusting BCI values for within-season variation using the historical dataset from the reference colony, the resulting standardized BCI (BCI_std_) values represent the expected body condition on the last sampling day of the season, April 14. This adjustment removed the confounding effect of sampling date progression, enabling direct spatial comparisons. We then used this standardized model to predict the expected BCI values for the specific days of the breeding season corresponding to our current sampling dates. The expected BCI reflects the condition an individual is predicted to have on a given day of the season, based on historical patterns. To quantify deviations from this expected condition, we calculated the difference between the observed and predicted BCI values:


$$ \mathrm{BC}{\mathrm{I}}_{\mathrm{deviance}}=\mathrm{BC}{\mathrm{I}}_{\mathrm{observed}}-\mathrm{BC}{\mathrm{I}}_{\mathrm{predicted}} $$

To test whether deviations in BCI differed significantly from the predicted value (i.e. whether the subtraction between observed and expected BCI equalled zero), we conducted one-sample *t* tests separately for each breeding locality and year (2024 and 2025).

To evaluate the effects of sex, sampling year, locality and NPP on pup body condition, we fitted linear models (LM) with BCI_std_ as the dependent variable. The global model included all predictors as fixed effects, as well as the interaction between locality and year to allow direct spatial and temporal comparisons. We also evaluated Gaussian GLMs and GAMs to test for potential non-linear effects of NPP, but these models did not improve fit and did not reveal any significant non-linear relationships; therefore, we retained the linear modelling framework.

Model selection followed an information–theoretic approach based on Akaike’s Information Criterion corrected for small sample size (AICc) (Burnham and Anderson, 2002), using the “dredge” function in the *MuMIn* package (Barton and Barton, 2015). All possible subsets of the global model were ranked by AICc, and the best-supported models were identified as those with the lowest AICc and ΔAICc < 2.

All data analyses were conducted using R version 4.4.1 (R Core Team, 2024) with statistical significance set at *P* < 0.05.

## Results

### Seasonal trend in pup body condition at the reference colony

**Figure 2:**
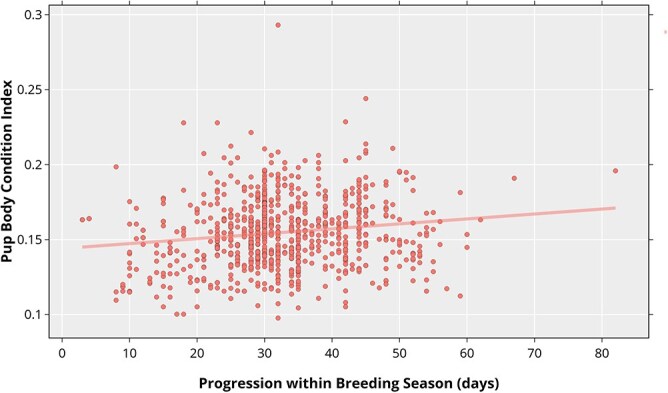
Relationship between body condition index (BCI) and progression into the breeding season in pups of South American sea lions (*O. flavescens*) from the historical dataset at Cobquecura (2009–2020, *n* = 834 pups). BCI was calculated as the ratio of body mass to standard length. The linear mixed-effects model fitted BCI as a function of days into the breeding season (“Progression”), with sex included as a random intercept. The model revealed a significant positive effect of progression on BCI, with an estimated increase of 0.000376 units per day.

The linear mixed-effects model, fitted with progression into the breeding season as a fixed effect and sex as a random intercept, revealed a significant positive relationship between BCI and progression (Fig. 2) in Cobquecura. Specifically, BCI increased by 0.000376 units per day (SE = 0.0000809, *t* = 4.65), indicating a gradual improvement in pup BCI as the breeding season advanced. Variation attributable to sex was negligible (variance = 8.50 × 10^−5^, SD = 0.0092) compared to the residual variance (variance = 5.82 × 10^−4^, SD = 0.0241). This slope value (*β*_1_ = 0.000376) was used to standardize the BCI values obtained in 2024 and 2025.

### Sex-based variation in morphometrics

Male South American sea lion pups were, on average, larger than females in all measured traits. Mean standard length was 89.7 ± 8.8 cm in males and 83.4 ± 7.6 cm in females, while mean axillary girth was 61.6 ± 10.2 and 56.9 ± 7.6 cm, respectively. Male pups were also heavier (17.0 ± 6.2 kg) than females (13.1 ± 4.8 kg) and exhibited a higher BCI (BCI: 0.185 ± 0.050; BCI_std_: 0.191 ± 0.047) compared to females (BCI: 0.154 ± 0.043; BCI_std_: 0.162 ± 0.040).

### Spatial variation in morphometrics

Overall, the largest pups were recorded in Isla Marta (Table 2), the southernmost site. This location showed the highest mean values for standard length, axillary girth and weight. In contrast, Cobquecura had the lowest mean standard length and weight, while Punta Lobos, the northernmost site, exhibited the lowest mean axillary girth.

**Table 2:** Mean ± SD of morphometric measurements of South American sea lion pups (*O. flavescens*) across colonies

**Colony**	**Mass (kg)**	**Axillary girth (cm)**	**Standard length (cm)**
Punta Lobos	12.94 ± 4.09	55.02 ± 6.03	84.65 ± 8.68
Isla Choros	13.33 ± 3.01	57.68 ± 4.81	85.20 ± 6.57
Cobquecura	11.77 ± 1.85	55.52 ± 3.72	82.37 ± 5.76
Isla Metalqui	14.10 ± 2.60	55.16 ± 5.08	85.13 ± 5.23
Isla Marta	25.75 ± 3.98	75.46 ± 7.59	99.85 ± 5.34

BCI_std_ varied among localities, with the highest mean values observed in Isla Marta (0.259 ± 0.031), followed by Isla Metalqui (0.172 ± 0.020), Isla Choros (0.162 ± 0.025), Punta Lobos (0.157 ± 0.029) and Cobquecura (0.151 ± 0.020).

### Drivers of body condition

The best-supported LM for pup BCI_std_ included locality, sex, year and the locality × year interaction (Table 3). Type II ANOVA revealed strong effects of locality (*F*₄,_146_ = 117.9, *P* < 0.001) and sex (*F*₁,_146_ = 17.3, *P* < 0.001), whereas year had no significant main effect (*F*₁,_146_ = 2.3, *P* = 0.130). A significant locality × year interaction (*F*₄,_146_ = 3.9, *P* = 0.005) indicated that spatial differences in BCI_std_ were not consistent across years. Importantly, NPP was not retained in any of the top-ranked models, suggesting no detectable effect of large-scale productivity on pup condition.

**Table 3:** Model selection results for standardized body condition index (BCI_std_) of South American sea lion (*O. flavescens*) pups

**Model**	***df***	**LogLik**	**AICc**	**ΔAICc**	**Weight**
Locality + Sex + Year + Locality:Year	12	380.771	−735.4	0	0.468
Locality + Sex + Year	8	372.814	−728.7	6.72	0.016
Locality + Sex	7	371.694	−728.6	6.74	0.016

For each model, the table shows the number of parameters (*df*), log-likelihood (LogLik), AICc, difference in AICc relative to the best model (ΔAICc) and model weight.

**Figure 3:**
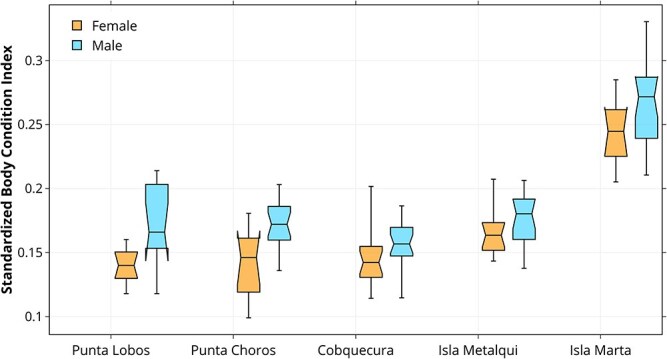
Standardized body condition index (BCI_std_) of male and female South American sea lion (*O. flavescens*) pups across five breeding locations along the Chilean coast. Pups from Isla Marta and Isla Metalqui exhibited significantly higher BCI_std_ than those from all other colonies. Across all sites, male pups had significantly higher BCI_std_ than females.

Parameter estimates confirmed these patterns. Pups from Isla Marta exhibited the highest BCI_std_, with values significantly greater than those from Cobquecura (*β* = 0.108 ± 0.007, *P* < 0.001). Pups from Isla Metalqui also showed higher values than Cobquecura (*β* = 0.025 ± 0.007, *P* < 0.001), whereas no significant differences were detected for Isla Choros (*β* = 0.003 ± 0.007, *P* = 0.65) or Punta Lobos (*β* = −0.004 ± 0.007, *P* = 0.54). Male pups had consistently higher BCI_std_ than females across all sites (*β* = 0.016 ± 0.004, *P* < 0.001) (Fig. 3).

**Figure 4:**
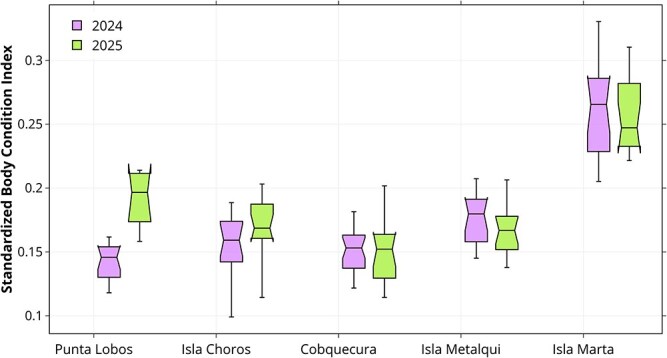
Standardized body condition Index (BCI_std_) of South American sea lion (*O. flavescens*) pups across five breeding locations along the Chilean coast, separated by year. Isla Marta and Isla Metalqui pups had significantly higher BCI_std_ than those from all other colonies in both years. A significant locality × year interaction was detected, driven mainly by the increase in BCI_std_ at Punta Lobos in 2025 compared to 2024.

Regarding temporal effects, year alone was not significant (*β* = 0.002 ± 0.007, *P* = 0.83). However, the interaction with locality revealed a marked increase in BCI_std_ at Punta Lobos in 2025 relative to 2024 (*β* = 0.037 ± 0.013, *P* = 0.004). Interaction terms for Isla Marta, Isla Metalqui and Isla Choros were not significant (all *P* > 0.25), indicating consistent year-to-year patterns in those colonies (Fig. 4). Overall, these results highlight locality and sex as the primary determinants of pup body condition, with Isla Marta consistently outperforming other sites, while Punta Lobos exhibited notable interannual variability.

### Difference from predicted BCI

**Figure 5:**
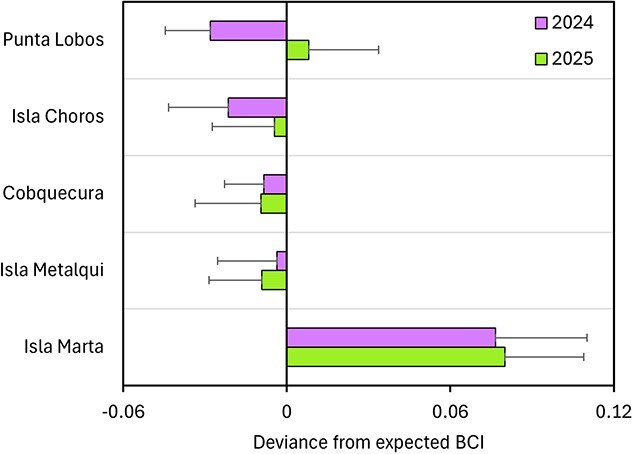
Deviance from the expected body condition index (BCI) of South American sea lion (*O. flavescens*) pups across five breeding localities along the Chilean coast in 2024 and 2025. Positive values indicate better-than-expected condition for a given day within the breeding season, whereas negative values indicate poorer-than-expected condition. Bars represent mean ± standard deviation.

Patterns of deviation from expected BCI varied among breeding localities and between years. The one-sample *t* tests revealed that deviations of BCI_std_ differed significantly from zero in all localities and years (all *P* < 0.001), but the magnitude and direction of deviations varied across sites. Isla Marta exhibited the highest positive deviations in both 2024 and 2025, indicating consistently better-than-expected pup condition. In contrast, Punta Lobos showed the largest negative deviation in 2024 but shifted to a slight positive deviation in 2025. Intermediate patterns were observed in Isla Choros, Cobquecura and Isla Metalqui, with mean deviations close to zero and limited interannual variation (Fig. 5).

## Discussion

Our study provides the first integrative assessment of spatial and temporal variation in pup body condition of South American sea lions along the Chilean coast. After correcting for within-season changes, we found that locality and sex were the strongest predictors of BCI_std_, while year and productivity had no significant effect. Male pups consistently exhibited higher body condition than females across all sites. This pattern is consistent with their higher energetic requirements, being met by greater maternal investment (Trivers and Willard, 1973; McMahon *et al.*, 2017; Séon *et al.*, 2025). Spatial differences were pronounced: the southernmost colonies, Isla Marta and, to a lesser extent, Isla Metalqui, supported pups in significantly better condition than the rest of the colonies, whereas Cobquecura, Isla Choros and Punta Lobos showed lower or intermediate values. Although broad-scale NPP did not explain variation in pup condition, the significant interaction between locality and year revealed that spatial differences were not entirely consistent across sampling years, with Punta Lobos in particular showing interannual variation. These findings highlight the combined influence of colony location, local environmental conditions and intrinsic factors, such as sex, in shaping early-life body condition in this species.

Spatial variation in BCI_std_ was substantial, with Isla Marta standing out as the colony with the highest pup condition. While females at Isla Choros are known to forage close to the colony (Muñoz *et al.*, 2014), little is known about foraging trip duration and distance in southern colonies. It is plausible that higher-latitude rookeries benefit from richer foraging grounds, allowing females to sustain higher milk output and thereby enhancing pup growth. Maternal effects are also likely to contribute, since pup mass and condition are strongly linked to maternal body length and foraging performance (Postma *et al.*, 2013; Desprez *et al.*, 2014; Séon *et al.*, 2025). Isla Marta, located within the Strait of Magellan, offers a unique oceanographic setting where Pacific, Atlantic and Southern Ocean waters converge (Panella *et al.*, 1991; Andrade *et al.*, 2025). This system, driven by complex marine–terrestrial–atmospheric interactions, sustains highly productive ecosystems (Iriarte *et al.*, 2007). In particular, squat lobsters (*Munida gregaria* and *M. surugosa*) dominate regional biomass (Castro *et al.*, 2021) and form a known component of the South American sea lion diet (Haro *et al.*, 2022), potentially providing a reliable and energy-rich prey base. If such prey resources are consistently available, females may enlarge cumulative advantages in body condition and foraging efficiency across years, potentially supporting higher and more stable maternal investment (Bradshaw *et al.*, 2004). This could help explain why Isla Marta pups consistently outperform those from other colonies, even when broad-scale productivity proxies show limited explanatory power. Such favourable ecological conditions, combined with maternal effects, may explain why pups from Isla Marta consistently outperform those from other colonies in terms of body condition.

Two other non-exclusive hypotheses may explain the latitudinal gradient in pup BCI_std_. First, the Rosenzweig productivity hypothesis predicts a positive relationship between environmental productivity and body size (Rosenzweig, 1968; Wigginton and Dobson, 1999). Although NPP did not emerge as a predictor in our models, local prey availability and foraging trip duration, which are not well captured by satellite-derived productivity, may better explain spatial variation (McCafferty *et al.*, 1998; Muñoz *et al.*, 2014; Sepúlveda and Harcourt, 2021). Second, Bergmann’s rule predicts that larger body sizes are favoured at higher latitudes and colder environments because larger bodies conserve heat more efficiently (Sepúlveda *et al.*, 2013; Goldenberg *et al.*, 2022). Empirical support for this pattern has been reported in both intraspecific (e.g. Sepúlveda *et al.*, 2013; Riemer *et al.*, 2018) and interspecific comparisons in mammals (e.g. Torres-Romero *et al.*, 2016; He *et al.*, 2023). In pinnipeds, however, within-species latitudinal gradients are often mixed; some studies report a Bergmannian pattern (Brunner, 2000; Sepúlveda *et al.*, 2013), whereas others do not (Brunner, 2000). Such inconsistencies may arise because local ecological conditions (e.g. resource availability), genetic divergence and sex-specific selection may override purely thermoregulatory effects (Goldenberg *et al.*, 2022). In our study, Isla Marta pups were the largest, consistent with the prediction that thermoregulatory constraints favour larger body sizes in colder habitats. Sepúlveda *et al.* (2013) tested Bergmann’s rule in adult South American sea lions by measuring the condylobasal length of skulls across a latitudinal gradient. Although they found that males and females do not follow Bergmann’s rule equally—with females appearing more influenced by productivity than temperature—both males and females from Punta Arenas, a locality near Isla Marta, were among the largest individuals recorded in the study. Third, large differences in colony size among rookeries could generate density-dependent effects through increased intraspecific competition for local prey, potentially influencing maternal foraging performance and pup condition (Bradshaw *et al.*, 2000). However, the absence of a monotonic relationship between colony size and pup BCI across our sites (e.g. high BCI at both Isla Marta and Isla Metalqui despite contrasting colony sizes) suggests that density dependence alone cannot explain the observed gradient.

The particularly high BCI_std_ values at Isla Marta may reflect both ecological and demographic processes. On the one hand, southern colonies may provide access to productive feeding grounds, allowing females to sustain higher milk output and pups to attain better condition (Riofrío-Lazo and Páez-Rosas, 2021). On the other hand, selective mortality could accentuate the pattern, as pups in poorer condition may have lower survival probabilities and thus be underrepresented in later-season samples (Oosthuizen *et al.*, 2015). Because Isla Marta was sampled relatively late in the season (March–April), the consistently higher BCI may partly reflect survival bias, with only the fittest pups remaining available for sampling. Although our design does not allow us to disentangle this effect from ecological drivers, pups from Isla Marta still deviated markedly from the expected BCI for that stage of the breeding season (Figs 3 and 4), standing out more than any other colony. In fact, in both years, Isla Marta exhibited the highest positive deviations, with mean values more than seven times greater than those observed at Punta Lobos in 2025. Together, these results suggest that while survival bias may contribute, ecological conditions and maternal effects are playing a role in shaping spatial patterns of pups’ body condition.

Our results revealed a significant interaction between locality and year, indicating that BCI_std_ differed between 2024 and 2025 in some, but not all, colonies. As shown in Fig. 4, pups from the northern colonies (Punta Lobos and Isla Choros) exhibited lower body condition in 2024 compared to 2025. In 2024, body condition in these colonies was much lower than expected, whereas in 2025, it was only slightly lower than expected at Isla Choros, and even higher than expected at Punta Lobos (Fig. 5). Because maternal investment and pup body condition can vary with prey availability, which is spatially and temporally heterogeneous (Goldsworthy, 2006; Sepúlveda *et al.*, 2014), these results suggest that 2024/2025 was a relatively “normal” year, in which mothers were able to compensate for resource fluctuations, whereas 2023/2024 was a comparatively poorer year, particularly in northern Chile. This pattern is consistent with the idea that females in consistently productive systems may accumulate long-term ecological advantages (e.g. familiarity with reliable foraging habitats) that enhance foraging efficiency across years, even when conditions are not uniformly favourable. These carryover effects could increase resilience to interannual shocks, whereas colonies relying on more variable prey fields may exhibit stronger year-to-year variation in pup condition (Bradshaw *et al.*, 2004; McMahon *et al.*, 2017). Interannual variability in pup condition in the northern colonies may be explained, at least in part, by the influence of El Niño/Southern Oscillation (ENSO). Otariids in the Pacific Ocean are known to be strongly influenced by periodic ENSO events, which affect prey abundance and availability (Trillmich and Limberger, 1985; Gentry, 1998). From July 2023 to May 2024, a strong ENSO event developed in the tropical Pacific, with sea surface temperatures rising sharply from 0.44°C below average in February 2023 to more than 2.4°C above average in October 2023 (World Meteorological Organization, 2023; Geng *et al.*, 2024). In northern Chile, ENSO events have been shown to alter the distribution, reproductive activity and biomass of anchovy (*Engraulis ringens*) (Hernández-Santoro *et al.*, 2019), one of the main prey items of the SASL in this region (Sielfeld *et al.*, 2018; Sarmiento-Devia *et al.*, 2020).

Two additional factors may also help explain the observed pattern at Punta Lobos. First, sampling dates differed between years by nearly a month: in 2024, pups were measured on February 29 and March 1, whereas in 2025, sampling took place on March 26, ~26 days later. Although BCI values were standardized to control for within-season effects, later captures could still have introduced a bias if pups in poorer condition were less likely to survive. Second, in 2025, only male pups were captured in Punta Lobos, and since males consistently exhibited higher BCI than females across all colonies, this sex bias in the sample may have inflated mean values at this site. Thus, the sharp reduction in pup condition observed in 2024 at Punta Lobos may reflect the combined effects of ENSO-related prey scarcity, sampling bias and sex-specific differences in pup body condition.

During both years of study and across all localities, male pups exhibited better body condition than females, a pattern consistent with early sexual size dimorphism reported in multiple otariids, including California sea lions (*Zalophus californianus*; Luque and Aurioles-Gamboa, 2001), Antarctic fur seals (*Arctocephalus gazella*; Lunn and Arnould, 1997), Steller sea lions (*Eumetopias jubatus*; Keogh *et al.*, 2013) and Australian fur seals (*Arctocephalus pusillus doriferus*; Arnould and Hindell, 2002; Wall *et al.*, 2023). In several otariid species, higher growth rates in males have been attributed to greater efficiency of energy use; although both sexes ingest similar amounts of milk relative to body mass, males gain mass more efficiently (Ono and Boness, 1996; Arnould and Hindell, 2002). However, in other cases, such as Antarctic fur seals, male and female pups grow at similar rates during lactation, with size dimorphism primarily reflecting differences present at birth (Lunn and Arnould, 1997; Keogh *et al.*, 2013). Studies of body composition in Steller sea lions and Australian fur seals indicate that such dimorphism may not only be a consequence of growth rate but also of sex-specific allocation strategies, with males tending to invest more in lean tissue while females accumulate proportionally greater lipid reserves (Arnould and Hindell, 2002; Keogh *et al.*, 2013). Overall, these findings suggest that sexual dimorphism in otariid pups is an early-life trait shaped by both differential growth efficiencies and distinct energetic allocation patterns between the sexes.

One limitation of this study is that sampling dates were not uniform across localities and years because of the weather and logistical constraints. Because pups grow continuously after birth, measurement timing could influence BCI and confound spatial comparisons. The historical dataset revealed a gradual increase in BCI as the breeding season advanced. This positive seasonal trend could reflect improved maternal foraging efficiency, increased milk transfer later in the season, or both (Oosthuizen *et al.*, 2019). Regardless of the underlying mechanism, these findings underscore the importance of accounting for temporal variation, such as seasonal progression, when comparing body condition among sites or years. However, empirical evidence in other otariids suggests that pup body condition remains relatively stable during the breeding period. For example, in Steller sea lions, a BCI applied to pups aged between 5 and 38 days showed no relationship with age, indicating minimal change in condition during the early postnatal period (Keogh *et al.*, 2013). Overall, the remarkable latitudinal differences in body condition among some localities collectively support the idea that, although pups exhibit continuous growth, short-term differences in sampling dates during the pupping season are unlikely to introduce systematic bias in comparisons of body condition among colonies—especially when adjustments, such as using a long-term reference dataset (e.g. Cobquecura), are applied.

A second limitation of our study was the low sample size relative to the size of the rookeries, which was due to permitting constraints rather than study design. To compensate for this limitation, in both years, pups were captured using a randomized protocol across the full spatial extent of each colony, minimizing potential bias associated with sex, maternal status or intra-colony location. Random sampling designs have been shown to provide reliable estimates of population-level body condition even at modest sample sizes (Nagel *et al.*, 2021). Similar sample sizes have been widely used in pinniped studies to infer spatial and temporal patterns in pup condition and maternal performance (e.g. Trites and Jonker, 2000; Green *et al.*, 2010). Based on these considerations, we therefore consider our estimates to be representative of each rookery–year combination.

Overall, our findings demonstrate strong spatial and sex-related differences in the body condition of South American sea lions pups along the Chilean coast, with especially high values observed at Isla Marta. While these patterns likely reflect a combination of ecological and maternal effects, future research is needed to clarify the underlying mechanisms. In particular, quantifying the duration and frequency of maternal foraging trips across colonies would provide critical insights into the energetic trade-offs faced by lactating females. Linking maternal foraging effort with pup growth trajectories would allow a more direct assessment of the cost–benefit balance of prey acquisition and its consequences for pup condition and survival. These studies would deepen our understanding of pinniped reproductive ecology and strengthen the use of this species as a sentinel for environmental change.

## Data Availability

The data that support the findings of this study are available from the corresponding author upon reasonable request.
